# Preparing nursing homes for a second wave of coronavirus disease 2019 (COVID-19)

**DOI:** 10.1017/ice.2020.1283

**Published:** 2020-10-20

**Authors:** A. Rekha Murthy, Jennifer A. Hanrahan, Sonali D. Advani, Muhammad Salman Ashraf, John P. Mills, Lona Mody, David K. Henderson, Mary K. Hayden, David J. Weber, Sharon B. Wright, Hilary Babcock, Judith Guzman-Cottrill, Sarah D. Haessler, Clare Rock, Trevor Van Schooneveld, Corey Forde, Latania K. Logan, Anurag Malani

**Affiliations:** 1 Division of Infectious Diseases, Department of Medicine, Cedars-Sinai Medical Center, Los Angeles, California; 2 Division of Infectious Diseases, University of Toledo College of Medicine and Life Sciences, Toledo, Ohio; 3 Department of Internal Medicine, Duke University School of Medicine, Durham, North Carolina; 4 Division of Infectious Diseases, Department of Internal Medicine, University of Nebraska Medical Center, Omaha, Nebraska; 5 Division of Infectious Diseases, Department of Internal Medicine, University of Michigan Medical School, Ann Arbor, Michigan; 6 Veterans’ Affairs Ann Arbor Healthcare System, Ann Arbor, Michigan; 7 Clinical Center, National Institutes of Health, Bethesda, Maryland; 8 Rush University Medical Center, Chicago, Illinois; 9 Division of Infectious Diseases, University of North Carolina, Chapel Hill, North Carolina; 10 Beth Israel Deaconess Medical Center, Boston, Massachusetts; 11 Washington University School of Medicine, St Louis, Missouri; 12 Infection Prevention and Epidemiology Consortium of BJC HealthCare, St Louis, Missouri; 13 Oregon Health and Science University, Portland, Oregon; 14 UMass Medical School-Baystate, Springfield, Massachusetts; 15 Johns Hopkins University School of Medicine, Baltimore, Maryland; 16 University of Nebraska Medical Center, Omaha, Nebraska; 17 Queen Elizabeth Hospital, Christ Church, Barbados; 18 St Joseph Mercy Health System, Ann Arbor, Michigan for the SHEA Board of Trustees

## Background

Among deaths related to coronavirus disease 2019 (COVID-19) in the United States, 40% have been linked to nursing homes.^1,2^ Nursing homes commonly have limited access to infection prevention and control (IPC) experts, and they are set up so that IPC duties are performed by a nursing home staff member or an infection preventionist with other responsibilities and little protected time. During the COVID-19 pandemic, nursing homes have faced challenges including inadequate infrastructure to support isolation units, difficulties in securing timely diagnostic testing, high staff turnover, space limitations, personal protective equipment (PPE) shortages, and frequently evolving guidance for prevention and treatment of COVID-19. This report outlines suggested models for collaboration, configuration, and controls to facilitate optimal preparedness and response for nursing homes during this pandemic and beyond.

## Collaboration

Local healthcare collaborative relationships are essential for nursing homes, and each participant and the broader community benefits. Whether maintained on an ongoing or as-needed basis, collaboratives provide support and expertise to coordinate approaches, bridge supply and resource gaps, prepare for patient influxes (eg, from a nursing home to hospital), and lessen strain across the community.

Ideally, healthcare collaboratives should include local nursing home facilities, local and state public health departments, health systems and hospitals, IPC and infectious diseases experts, and laboratories. Also, they should be able to expand. Healthcare collaboratives are best if built before a crisis, but in places where collaboratives do not exist, we encourage local healthcare communities to form them now.

## Configuration

State and local public health departments are best positioned to initiate collaboratives. They are informed in resource-sharing efforts and available funding, and they can expand capacity by partnering with academic medical centers and hospital systems. Also, IPC experts and healthcare epidemiologists can work on behalf of public health to establish consulting structures with nursing homes. We recommend that participants in the collaborative formalize the relationship with a written agreement. At minimum, the agreement should establish the following:

- Buy-in from each facility’s leadership
- Standards that foster trust, supporting mutual problem solving and transparency, and providing protection against punitive actions
- Roles and responsibilities of participants.

Activities of the collaborative may include the following:

- Providing access to IPC experts when a COVID-19 case is identified in a nursing home
- Establishing preferred lines of communication for routine prevention, outbreaks, and crises
- Creating protocols for nursing homes to alert public health to shortages of supplies or capacity and for public health to coordinate sourcing
- Coordinating transfers of residents with COVID-19 out of nursing homes that are unable to safely care for them.

## Controls

To accomplish these goals, the collaborative should reinforce adherence to IPC standards^3^ and designate staff members responsible for training and monitoring adherence by nursing home staff.

### Symptom screening

Signs and symptoms of COVID-19 in nursing home residents and staff members must be identified early. A single case of COVID-19 in the nursing home should prompt escalation in IPC procedures,^4^ testing, and exposure evaluations.^5,6^

All residents, staff, and visitors who are allowed in the facility should undergo daily temperature and symptom monitoring. Human resources policies should be established for staff reporting, monitoring, and returning-to-work, and staff must be educated regarding the symptoms of COVID-19, including that temperature monitoring alone is insufficient.

### Testing

Healthcare personnel (HCP) performing testing should wear appropriate PPE and should be trained in specimen collection. Because of ongoing supply issues, nursing homes may decide to implement >1 type of test. HCP should be trained accordingly.

The following factors should be considered when choosing tests:

- Sensitivity/specificity
- Turnaround time
- Availability of specimen collection and test supplies
- Cost
- Comfort. Nasopharyngeal (NP) swab collection can be uncomfortable and test refusal may become an issue. Other methods, such as oropharyngeal (OP) or anterior nares swabs may offer more comfort with comparable sensitivity.

We do not recommend for or against use of antigen testing in the nursing home population because of limited scientific information at this time. The Centers for Disease Control and Prevention (CDC) and the Centers for Medicare and Medicaid Services (CMS) released guidance on antigen testing in nursing homes, and the CMS is sending antigen testing kits to nursing homes with the expectation that they will be used to test asymptomatic staff.^7^ These tests have not been validated for screening asymptomatic individuals yet. Nursing homes should be informed about test performance and perform confirmatory tests when needed. Considerations may include reported sensitivity and specificity of the test, pretest probability of infection in the person tested (eg, compatible symptoms, known exposures), and prevalence of COVID-19 in the facility or community.

Nursing homes should test often, but frequency may change based on the type of test, rates of community transmission, and number of cases in the facility. Before relaxing mitigation strategies, CMS currently requires the following:

- Baseline testing of all residents
- Baseline and periodic testing for all staff, volunteers, and vendors
- Written protocols for testing, including actions for individuals who refuse or cannot consent to testing
- Arrangements with laboratories for timely test turnaround.^8^

The CDC no longer recommends repeat testing to discontinue isolation of individual patients.^9^ Residents with COVID-19 can be removed from isolation after 10 days from symptom onset if at least 24 hours have passed since the last fever without use of antipyretics, and with improvement in symptoms, or after 20 days for severely ill or immunocompromised individuals.^9^ We do not recommend that nursing homes require negative tests for discharge or transfer from the hospital.

### Cohorting, isolation, and contact tracing

A nursing home with an identified case of COVID-19 consequently has the following:

- Residents and/or staff with COVID-19 (suspected or confirmed)
- Exposed and asymptomatic residents and/or staff
- Unexposed residents and/or staff.

The nursing home infection preventionist should be familiar with the process of contact tracing and should be able to liaise with public health to identify residents and staff who were exposed to the positive case.

The CDC guidance calls for caring for residents with COVID-19 in a dedicated unit with dedicated HCP^10^; however, cohorting and isolation in nursing homes can be complex due to facility design, staffing shortages, and limited isolation rooms. A healthcare collaborative can help nursing homes prepare for and manage this. Nursing homes should have a cohorting and isolation plan that does the following:

- Identifies a location for management of residents with COVID-19 (suspected or confirmed). If a nursing home cannot designate a whole unit or a section of a unit, it should assign dedicated staff to these residents with clear signage on rooms
- Prepares a staffing plan for consistent and dedicated staff for each cohort
- Documents IPC procedures and protocols for each type of cohort, including the following, as appropriate:
  - Isolation/quarantine
  - PPE use
  - Environmental cleaning/disinfection
  - Engineering controls
  - Audit and feedback processes
- Describes contingencies, for example, increases in COVID-19 cases and staffing shortages.

### Personal protective equipment (PPE)

Appropriate use of PPE by HCP, staff, residents, and visitors helps prevent the transmission of COVID-19 in nursing homes (Table 1).

**Table 1.** Use of Personal Protective Equipment

Measure	HCP	Non-HCP staff	Residents	Visitors
Universal precautions (at all times, in all spaces of facility and grounds)	Medical mask Consider eye protection	Medical mask, if interacting with resident(s) Medical mask or cloth mask acceptable, if not interacting with resident(s)	If tolerated, medical mask preferred when interacting with HCP, staff, residents, or visitors, or cloth mask acceptable	Medical mask, if interacting with resident(s) Medical or cloth mask, if not interacting with resident(s)
Ongoing community transmission and/or new/first case detected in nursing home	Medical mask Eye protection Gown Gloves			
Care of resident with COVID-19	Medical mask or N95 (if supplies permit) Eye protection Gown Gloves			Medical mask (eg, end-of-life visit),
AGP on resident with COVID-19	N95 Eye protection Gown Gloves	N/A		
AGP on resident without COVID-19	Medical mask or N95 (if supplies permit) Eye protection Gown Gloves			

Note. HCP, healthcare personnel; AGP, aerosol-generating procedure.

### Universal precautions

Practice universal precautions during the pandemic for source control. They apply to the following:

- All nursing home staff members should wear face coverings. For HCP, this includes:
  - Medical masks
  - Eye protection, in settings with moderate to substantial community transmission. It is considered optional for settings with minimal to no community transmission, unless otherwise indicated as part of standard precautions.^11^ When worn at all times except in a private office, universal eye protection may be used as a method to prevent HCP-to-HCP transmission.
- Residents should wear face coverings, if tolerated, when interacting with HCP, staff, residents, or visitors. When feasible, medical masks are preferred.
- Visitors allowed in the facility should wear face coverings. A visitor in direct contact with resident(s) should wear a medical mask rather than a cloth mask.

### Transmission-based precautions

For direct care of residents with COVID-19 (suspected or confirmed), HCP should wear medical masks, eye protection, gowns, and gloves.^11,12^ HCP may alternatively wear fit-tested N95s instead of medical masks^11,12^; however, many nursing homes face limitations in maintaining fit testing programs, and when supplies are constrained, N95s should be reserved for HCP performing aerosol-generating procedures (AGPs) as outlined below.

### Precautions for aerosol-generating procedures

When performing an AGP (eg, use of a nebulizer, bilevel positive airway pressure, or continuous positive airway pressure) on a resident with COVID-19 (suspected or confirmed), HCP should wear an N95 or equivalent, eye protection, gown, and gloves. In settings with ongoing or widespread community transmission, consider this combination for all AGPs regardless of a resident’s COVID-19 status, if supplies permit.

## Maintaining supply and managing shortages

Apply a multipronged approach to optimize PPE and avoid shortages:

- Designate staff member(s) to do the following:
  - Steward PPE supplies, including use of a PPE burn rate calculator
  - Monitor donning and doffing
  - Provide feedback
- Bundle resident care activities to minimize entries into residents’ rooms
- Establish policies to extend use of, to extend the reuse of, and to decontaminate PPE.

## Ventilation

Know about the building’s ventilation system, including the HVAC filter level (current and highest level achievable), outdoor air-dampener settings, areas with negative or positive pressure, the number of air changes per hour in rooms and common spaces, and when and how long the system runs.^13^ Professional evaluation may be needed to determine air circulation patterns, risk of recirculated air, and whether air travels between areas with residents with COVID-19 (suspected or confirmed), other residents’ spaces or rooms, and staff areas.

## Staffing

All nursing home staff members are potential vectors of COVID-19, especially during periods of active community transmission. They often work at multiple facilities, increasing the risk of intra- and interfacility spread. Many nursing homes experience high staff turnover, leading to a less experienced workforce and employment of ancillary staff or volunteers. Reports of nursing home outbreaks of COVID-19 identified staff who worked while symptomatic and inadequate knowledge of IPC precautions as potential causes of transmission.^14^

The CDC recommends at least one full-time IP for nursing homes with >100 residents and/or nursing homes with ventilators or hemodialysis capabilities.^12^ nursing home administration should provide its infection preventionist(s) with dedicated, protected time.

Nursing homes should also, regardless of size, provide IPC education for at least 1 full-time staff member annually, and training in IPC practices for all staff.^12^

## Physical distancing

Outbreaks in nursing homes also have been traced to staff interactions in non–resident care areas. Staff should wear masks in all shared work rooms, break rooms, administrative offices, and nursing stations when in the presence of another person in that space, and take meals and snacks alone or outside with appropriate distancing, or add engineering controls (eg, room dividers, plexiglass). Physical distancing should be practiced, especially while eating or drinking.

In conclusion, the COVID-19 pandemic has led to unprecedented challenges for nursing homes. Anticipating a second wave, we urge the creation of formalized collaborative relationships between nursing homes, public health, and local hospitals and labs. In addition, the United States needs a national strategy for resource allocation, program development, management, and staff and patient protection in partnership with local and state health departments to increase funding, reporting, and regulation for nursing homes.
